# Astaxanthin Supplementation Effects in Right Ventricle of Rats Exposed to Chronic Intermittent Hypobaric Hypoxia

**DOI:** 10.3390/antiox13101269

**Published:** 2024-10-18

**Authors:** Eduardo Pena, Samia El Alam, Constanza Gonzalez, Isaac Cortés, Diego Aguilera, Karen Flores, Karem Arriaza

**Affiliations:** 1 High Altitude Medicine Research Center (CEIMA), Arturo Prat University, Iquique 1100000, Chile; selalam@unap.cl (S.E.A.); conigonzol@gmail.com (C.G.); diego.aguilera.a@gmail.com (D.A.); kfloresu@unap.cl (K.F.); karriza@unap.cl (K.A.); 2 Science Faculty, Arturo Prat University, Iquique 1100000, Chile; isacortes@unap.cl

**Keywords:** altitude hypoxia, oxidative stress, nutraceutical, hypertrophy, right ventricle

## Abstract

In Chile, individuals are commonly exposed to high altitude due to the work shift system, involving days of exposure to high altitude followed by days at sea level over the long term, which can result in chronic intermittent hypobaric hypoxia (CIHH). CIHH can cause high-altitude pulmonary hypertension (HAPH), the principal manifestation of which is right ventricular hypertrophy (RVH), in some cases leading to heart failure and eventually death. Studies have shown the contribution of oxidative stress and inflammation to RVH development. Recently, it was determined that the pigment astaxanthin has high antioxidant capacity and strong anti-inflammatory and cardioprotective effects. Therefore, the aim of this study was to determine the effects of astaxanthin on RVH development in rats subjected to CIHH. Methods: Thirty two male Wistar rats were randomly assigned to the following groups (n = 8 per group): the normoxia with vehicle (NX), normoxia with astaxanthin (NX + AS), chronic intermittent hypobaric hypoxia with vehicle (CIHH), and chronic intermittent hypobaric hypoxia with astaxanthin (CIHH + AS) groups. CIHH was simulated by 2 days in a hypobaric chamber followed by 2 days at sea level for 29 days. Results: Exposure to CIHH induced RVH and increased lipid peroxidation (MDA), Nox2 expression, and SOD activity, however, it decreased pro-IL-1β expression. Astaxanthin restored oxidative stress markers (Nox2 and MDA), increased GPx activity, and decreased RVH compared to CIHH. Conclusion: Astaxanthin alleviates RVH and reduces Nox2 and MDA levels while increasing GPx activity in rats subjected to CIHH. These findings provide new insights of astaxanthin as a new nutraceutical against high-altitude effects.

## 1. Introduction

As altitude increases, atmospheric pressure decreases. For example, at sea level, the barometric pressure is 760 mmHg, but on Mount Everest, which is located 8849 m above sea level (m.a.s.l.), the barometric pressure decreases to 250 mmHg. High altitude is defined as an altitude above 2500 m.a.s.l. [1]. Notably, at this altitude, the partial pressure of oxygen decreases, causing a decrease in the amount of O_2_ that is transported by the blood to tissues and cells (hypoxia). Therefore, exposure to high altitude results in hypobaric hypoxia, which can cause physiological alterations in aerobic organisms [2,3].

There are different types of hypobaric hypoxia depending on the time of exposure. Exposure to high altitude for a short period (hours or days), which is commonly experienced by alpinists and tourists, is termed acute hypobaric hypoxia. Chronic hypobaric hypoxia is experienced by people who live permanently at high altitude. In Chile, individuals are particularly prone to hypobaric hypoxia because the majority of mining activities are carried out at high altitudes. Specifically, more than 20,000 people in Chile participate in 7 days of work at high altitude followed by 7 days of rest at sea level. This activity has been termed the “Chilean mining model of chronic intermittent hypoxia” [4], which corresponds to chronic intermittent hypobaric hypoxia (CIHH), but the condition is also of interest among other public and private entities.

The principal effects and pathologies of CIHH include acute mountain sickness (AMS) on the first day of a shift [5] and the development of high-altitude pulmonary hypertension (HAPH) [6], which is characterized by an increase in pulmonary artery pressure (≥30 mmHg) and may result in right ventricular hypertrophy (RVH) [2,6]. Moreover, if this compensatory hypertrophic mechanism is sustained over a long period, the right ventricle (RV) becomes dilated [7], leading to heart failure and ultimately death [8].

Several studies have proposed that oxidative stress is one of the principal contributors to cardiac hypertrophy [9,10]; moreover, studies have shown that exposure to hypoxia increases the level of reactive oxygen species (ROS) in many cell lines [11]. Specifically, studies in humans chronically (native) or acutely (48 h) exposed to high altitude have shown increased lipid peroxidation in plasma and 8-isoprostaglandin-F2α (8-iso-PGF2α) levels in urine [12] and have corroborated that oxidative stress is triggered by hypobaric hypoxia [13]. In fact, studies in cultured chicken cardiomyocytes have demonstrated that hypoxia increases the levels of oxidative stress biomarkers such as 4-hydroxynonenal (4-HNE) and malondialdehyde (MDA). A previous study on CIHH by our group revealed an increase in the levels of nicotinamide adenine dinucleotide phosphate (NADPH) oxidase, specifically the Nox2 subunit, the principal source of ROS, in the context of CIHH-induced RVH [14,15]. In addition, the levels of the mitogen-activated kinase p38 (p38 MAPK) and transcription factors such as nuclear factor kappa B (NF-κB) and hypoxia-inducible factor (HIF)-1 are increased under hypoxic conditions [14,15,16].

Another important factor involved in RVH and heart failure development is inflammation due to increases in the levels of interleukin (IL)-6, IL-1β, tumor necrosis factor-a (TNF-α), and NF-κB [17]. Studies have demonstrated increases in the levels of TNF-α, NF-kB, and IL-6, which are related to RVH [18] and the decompensated transition to right heart failure [14], under chronic hypobaric hypoxia. However, few studies have evaluated the inflammatory molecules involved in the development of RVH resulting from CIHH.

Recent evidence has shown that astaxanthin, which is a carotenoid pigment of the xanthophyll family with high antioxidant capacity and strong anti-inflammatory and cardioprotective effects, can alleviate cardiac hypertrophy [19]. Astaxanthin has been suggested to prevent cardiac hypertrophy and heart failure, possibly by limiting the generation of mitochondrial superoxides [20]. Several studies have shown that treatment with astaxanthin can reduce the levels of oxidative stress markers, normalize LV systolic pressure, ameliorate cardiac hypertrophy, and decrease lipid peroxidation in the heart. Moreover, astaxanthin can restore the activity of antioxidants, such as superoxide dismutase (SOD), catalase (CAT), and glutathione (GSH), in the rat heart, decrease the infiltration of inflammatory cells, and protect against isoprenaline-induced myocardial damage [21,22]. Furthermore, astaxanthin significantly suppresses Toll-like receptor 4 (TLR-4), NF-κβ, and TNF-α expression in cardiac tissue, increases miR-138 expression, and significantly decreases HIF-1α levels, thus protecting the myocardium [23,24]. Therefore, the aim of this study was to determine the effects of astaxanthin administration on right ventricular hypertrophy, oxidative stress, and inflammation in rats subjected to CIHH.

## 2. Materials and Methods

### 2.1. Animal Model and Study Groups

Thirty two male Wistar rats (3 months old) obtained from the High Altitude Medicine Research Center, Arturo Prat University, Iquique, Chile, were used in this study. All procedures and protocols involving the animals were approved by the Research Ethics Committee of Tarapacá University (N° 10/2023). Furthermore, this study was carried out in accordance with the ethical standards for the management of experimental animals (Chilean Law 20,380, Art 7, 3 October 2009).

The rats were housed in individual cages at a temperature of 22 ± 2 °C on a 12 h light/12 h dark cycle; the feeding consisted of 20 g/day of food containing 22.0% crude protein, 5.0% crude fiber, 9.0% ash, and 12% moisture (5POO^®^, LabDiet, Prolab RMH3000, Richmond, IN, USA) and water was provided ad libitum. No physical exercise was performed; however, movement within the cage was not restricted.

The rats were randomly divided into 4 experimental groups (*n* = 8): the normobaric normoxia (NX) group, which was used as a control (sea level) group; the normobaric normoxia plus astaxanthin (50 mg/kg/day; NX + AS) group; the CIHH group, which was housed for 2 days under hypobaric hypoxia followed by 2 days at sea level; and the CIHH plus astaxanthin administration (CIHH + AS) group, which was housed for 2 days under hypobaric hypoxia followed by 2 days at sea level, and treated with astaxanthin (50 mg/kg/day). The dose of astaxanthin (Atacama Bio Natural Products S.A., Iquique, Chile) was selected on the basis of previously published dose–response studies [25,26], and it was administered through voluntary ingestion using unflavored gelatin cubes [27,28]. For voluntary ingestion, rats were initially trained to accept unflavored gelatin without astaxanthin. The gelatin was placed in their cage for 2 h until the cube was completely consumed. This procedure was repeated for 3 to 4 days. Astaxanthin treatment was initiated at the beginning of the exposure to CIHH.

### 2.2. Hypobaric Hypoxia Exposure

The experimental protocol was carried out for 29 days for all groups, and hypobaric hypoxia was achieved with the use of a hypobaric chamber at 428 Torr, which is equivalent to a pressure at an altitude of 4600 m. The internal air flow in the chamber was 3.14 L/min, and the humidity was between 21 and 30%. The time necessary to reach the final pressure was 1 h. The rats in the NX group were maintained under the same environmental conditions but without hypobaric hypoxia exposure. The protocol used was based on a strategy that, despite the limitations of animal models, rigorously mimics long-term exposure to CIHH in humans [3,29]. On the last day (day 29), the rats were anesthetized with ketamine (90 mg/kg body weight) and subsequently sacrificed by fatal thoracotomy, and blood and heart samples were obtained. The blood, RV, left ventricle (plus septum), and urine samples were immediately frozen at −80 °C for subsequent molecular studies.

### 2.3. Biomedical Variables

At the beginning (day 0) and end (day 29) of the exposure period, the body weights (BWs; g) of the rats were measured via an electronic scale (Acclab V-1200^®^, Lake Country, IL, USA). The hematocrit value (Hct%), at the end of the protocol, was measured by transferring blood samples to a glass capillary tube and centrifuging them at 5000 rpm for 5 min at 4 °C (5804 R, Eppendorf AG^®^, Hamburg, Germany). The blood samples were obtained from rats in heparinized vials after fatal thoracotomy via puncture of the inferior vena cava.

### 2.4. Ventricular Hypertrophy

The rat heart was removed, and the RV was subsequently separated, weighed, immediately frozen, and stored at −80 °C. The occurrence of RVH was assessed via Fulton’s index (the ratio between the weight of the RV (g) and the weight of the left ventricle plus septum (g)) [30,31].

### 2.5. Lipid Peroxidation and 8-Isoprostane

Lipid peroxidation was evaluated in RV tissues by determining the malondialdehyde (MDA) concentration (μmol/L) via a colorimetric assay. First, 30 mg of RV tissue was homogenized in 300 μL of RIPA buffer (50 mM Tris-HCl, 1% Triton Pak^®^; Brinton, IL, USA) at 4 °C. Then, 100 µL of the sample was mixed with 200 µL of 10% trichloroacetic acid on ice for 30 min. The mixture was subsequently centrifuged at 4000 rpm for 15 min at 4 °C. Two hundred microliters of the supernatant was subsequently mixed with 200 µL of 0.67% thiobarbituric acid and incubated in a bath at 100 °C for 1 h. Finally, with the use of a spectrophotometer (Thermo Electron Corporation^®^, Madison, WI, USA), the absorbance was measured at 532 nm. To measure 8-Isoprostane (8-iso-PGF 2α) in urine samples, a colorimetric ELISA assay (ab175819; Abcam^®^, Cambridge, UK) was performed according to the manufacturer’s instructions.

### 2.6. CAT, GPX, and SOD Activity

To determine the antioxidant activity of CAT and GPx (ab83464; ab102530; Abcam^®^, Cambridge, UK) in RV tissue and that of SOD (EIASODC; Invitrogen™, Waltham, MA, USA) in plasma, colorimetric assays were performed according to the manufacturer’s instructions.

### 2.7. Western Blot Analysis

To obtain protein, 30 mg of RV tissue was homogenized with 300 µL of RIPA buffer containing phosphatase and protease inhibitors (1 mM PMSF, 1 µg/mL leupeptin, 5 mM EDTA, 1 mM EGTA, 10 mM NaF, and 1 mM DTT). The mixture was subsequently centrifuged (5804 R, Eppendorf AG^®^, Hamburg, Germany) at 12,000 rpm for 20 min at 4 °C, after which the supernatant was removed. The total protein concentration was quantified via the Bradford method [32] by measuring the absorbance at 590 nm with a microplate reader (Infinite^®^ 200 PRO, TECAN^®^, Männedorf, Switzerland). The proteins were stored at −80 °C.

For the immunoblot analysis, the protein samples were diluted with 2x Laemmli buffer (0.125 M Tris-HCl, 4% (*w*/*v*) SDS, 20% (*v*/*v*) glycerol, 0.004% bromophenol blue, and 10% β-mercaptoethanol, pH 6.8) and separated by molecular weight on sodium dodecyl sulfate–polyacrylamide gel electrophoresis (SDS–PAGE) and 30% bisacrylamide (*v*/*v*) gels. The proteins were subsequently transferred to a polyvinylidene fluoride (PVDF) membrane with a semidry electrotransfer system (OWL TM Separation Systems, Panther Semi-Dry Electroblotters, Thermo Scientific^®^, Barrington, IL, USA).

Then, the PVDF membrane was blocked with bovine serum albumin (BSA) and incubated with primary antibodies against gp91-phox/Nox2 (1:200, No. sc-130543, lot No. C0123, Santa Cruz Biotechnology^®^, Dallas, TX, USA), IL-6 (1:2000, No. sc-57315, lot. No. C1721, Santa Cruz Biotechnology^®^), TNF-α (1:500, No. sc-52746, lot No. B0821, Santa Cruz Biotechnology^®^), NF-κB (1:200, lot No. sc-8008, lot No. C2421, Santa Cruz Biotechnology^®^), IL-1b (1:500, No. ab234437, lot No. 1030745-21, Abcam), CAT (1:300, No. MA5-42573, lot No. ZF4361989A, Invitrogen), 4-HNE (1:500, No. bs-6313R, lot No. BB09163582, Bioss Antibodies, Woburn, MA, USA), and β-actin (1:5000, No. sc-47778 HRP, lot No. J1822, Santa Cruz Biotechnology^®^) overnight at 4 °C. The membrane was then incubated with a BP-HRP-conjugated anti-mouse m-IgGκ (1:2000, No. sc-516102, lot No. A2219, Santa Cruz Biotechnology^®^) and HRP-conjugated anti-rabbit IgG (1:2000, no. sc-2357, Lot No. C2818, Santa Cruz Biotechnology^®^) secondary antibodies diluted in 3% BSA for 1 h at room temperature. A chemiluminescence kit (Clarity^TM^ Western ECL Substrate, Cat. 170-5061, Bio-Rad, Hercules, CA, USA) was used to visualize the protein bands in the dark. An automatic imaging system (ImageQuant^®^ LAS 500 de GE Healthcare Life Sciences^®^, Uppsala, Switzerland) and ImageJ program 1.54k were used for normalization to the expression of β-actin and densitometric analysis of the bands.

### 2.8. Data Analysis

The data were analyzed via the R programming language [33]. Basic descriptive statistics such as the minimum, maximum, mean, and quartiles were first calculated for each variable. Then, the Shapiro–Wilk test and Welch t test were performed. For nonparametric values the Kruskal–Wallis test was used. The significance level was alpha = 0.05.

## 3. Results

### 3.1. Body Weight

**Figure 1:**
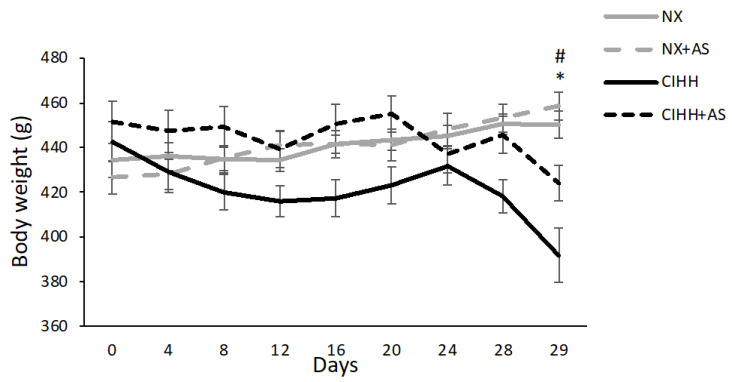
Body weight (BW; g) of normobaric normoxia (NX), normobaric normoxia plus astaxanthin administration (NX + AS), chronic intermittent hypobaric hypoxia (CIHH), and chronic intermittent hypobaric hypoxia plus astaxanthin administration (CIHH + AS) groups. The values are the means (x^−^) ± standard errors of the means (SEMs). * *p* < 0.05: hypobaric hypoxia groups vs. normoxia group; # *p* < 0.05: CIHH group vs. CIHH + AS group.

At the end of the protocol (day 29), BW was decreased in the CIHH and CIHH + AS groups compared to the NX and NX + AS groups (*p* < 0.05). However, the BW of the CIHH + AS group was greater than that of the CIHH group (*p* < 0.05) (Figure 1).

### 3.2. Hct%

**Figure 2:**
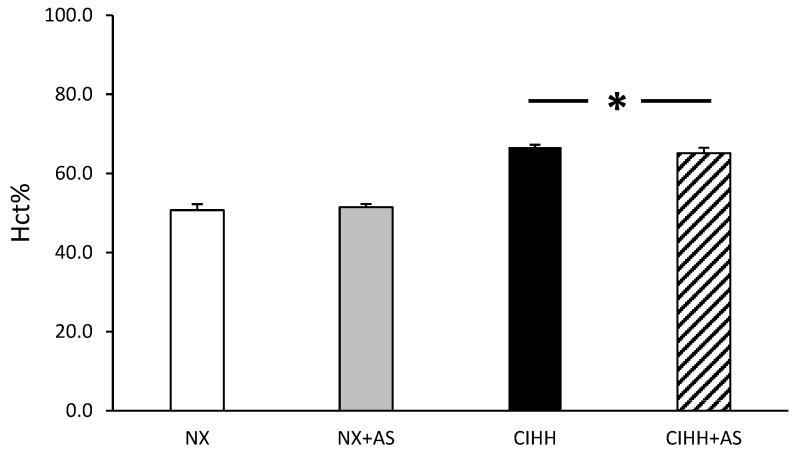
Hematocrit percentage (Hct%) of the normobaric normoxia (NX), normobaric normoxia plus astaxanthin administration (NX + AS), chronic intermittent hypobaric hypoxia (CIHH), and chronic intermittent hypobaric hypoxia plus astaxanthin administration (CIHH + AS) groups. The values are the means (x^−^) ± standard errors of the means (SEMs). * *p* < 0.05: hypobaric hypoxia groups vs. normoxia group.

At the end of the exposure period (day 29), the hypobaric hypoxia groups (CIHH and CIHH + AS) presented with a higher Hct% than the normoxic groups (*p* < 0.05), however, there were no differences between the CIHH groups (*p* = N.S.) (Figure 2).

### 3.3. RVH

**Figure 3:**
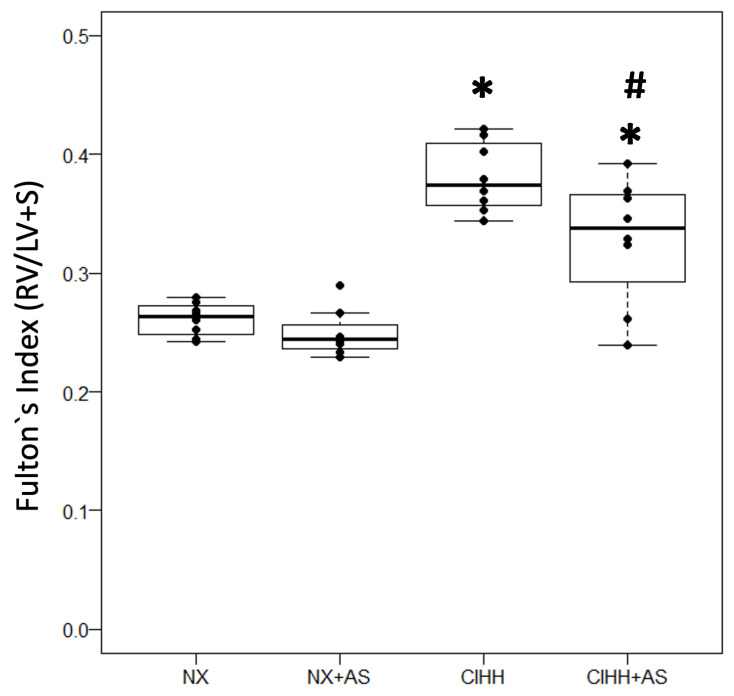
Right ventricular hypertrophy (RVH) was assessed via Fulton’s index (weight of right ventricle (RV)/weight of left ventricle plus septum) in the normobaric normoxia (NX), normobaric normoxia plus astaxanthin administration (NX + AS), chronic intermittent hypobaric hypoxia (CIHH), and chronic intermittent hypobaric hypoxia plus astaxanthin administration (CIHH + AS) groups. The values are the means (x^−^) ± standard errors of the means (SEMs). * *p* < 0.05: hypobaric hypoxia groups vs. normoxia group; # *p* < 0.05 CIHH + AS vs. CIHH group.

The rats in the CIHH group presented with significantly greater Fulton’s index values than those in the normoxic groups (NX and NS + AS) (*p* < 0.05), which indicated the presence of RVH. However, the rats in the CIHH + AS group presented with a decrease in Fulton’s index, being significantly different compared to the CIHH group (*p* < 0.05) (Figure 3).

### 3.4. Oxidative Stress Markers

**Figure 4:**
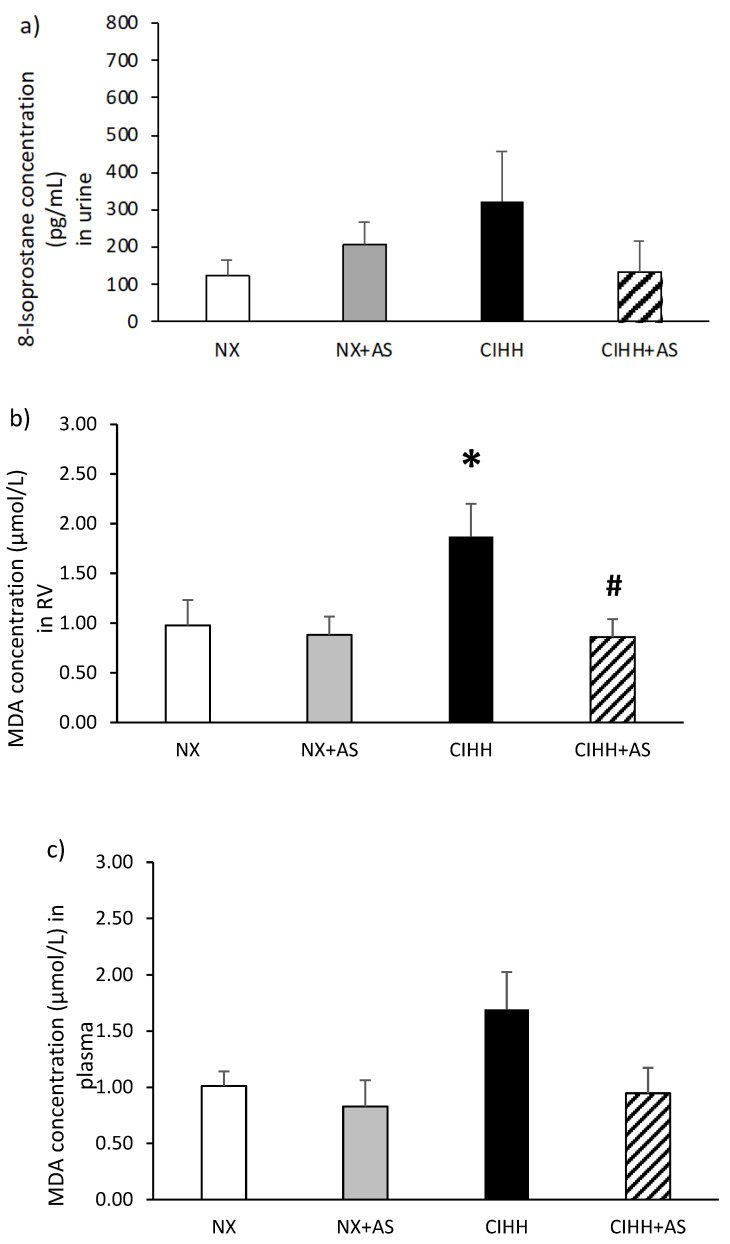

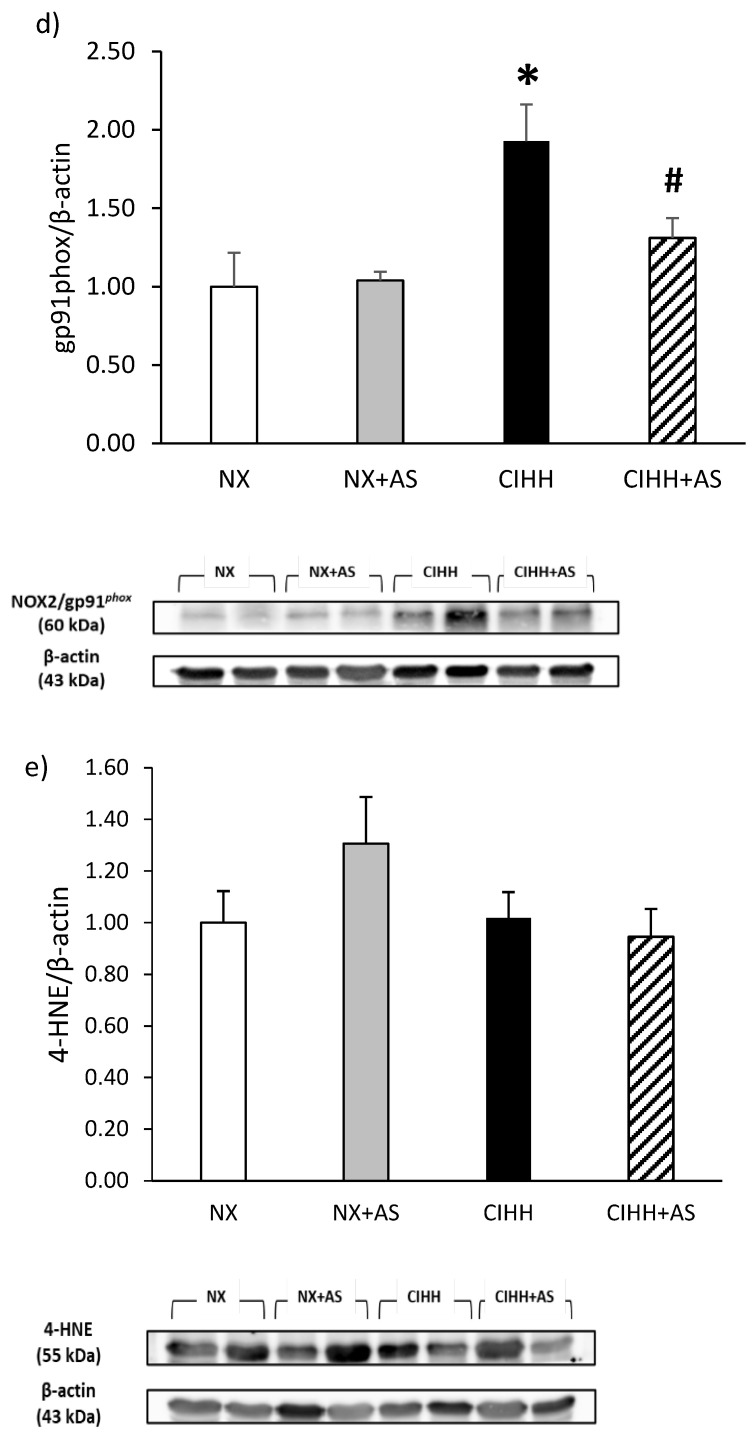
(**a**) 8-Isoprostane concentrations in the urine; (**b**) malondialdehyde (MDA) concentrations in right ventricle (RV) tissue; (**c**) MDA concentrations in plasma; (**d**) expression of nicotinamide adenine dinucleotide phosphate oxidase-2 (Nox2) in right ventricle (RV); (**e**) 4-hydroxynonenal (4-HNE) in right ventricle (RV). The rats in the normobaric normoxia (NX), normobaric normoxia plus astaxanthin administration (NX + AS), chronic intermittent hypobaric hypoxia (CIHH), and chronic intermittent hypobaric hypoxia plus astaxanthin administration (CIHH + AS) groups were compared. The values are the means (x^−^) ± standard errors of the means (SEMs). * *p* < 0.05 CIHH group vs. normoxia group; # *p* < 0.05 CIHH + AS vs. CIHH group.

The concentration of 8-isoprostane in the urine of the rats was not significantly increased in the CIHH group (*p* = N.S.; Figure 4a). The concentration of lipid peroxides, as indicated by the level of malondialdehyde (MDA), was greater in the RV tissue of the CIHH group than in that of the normoxic group (*p* < 0.05; Figure 4b). Notably, the administration of astaxanthin decreased lipid peroxidation in the RV of rats exposed to hypobaric hypoxia (the CIHH + AS group), returning it to the level of those in the normoxic control group. The plasma MDA concentration was not significantly greater (*p* > 0.05) in the CIHH group than in the other groups (Figure 4c). Regarding the Nox2 expression in the RV, it was increased in rats in the CIHH group compared with those in the NX group. Moreover, the rats in the CIHH + AS group presented with a decrease in the expression of Nox2, which returned to the control level (*p* < 0.05; Figure 4d). Finally, 4-HNE levels in the RV did not significantly differ between the groups (*p* = *NS*; Figure 4e).

### 3.5. CAT, Glutathione Peroxidase (GPx) and SOD Levels

**Figure 5:**
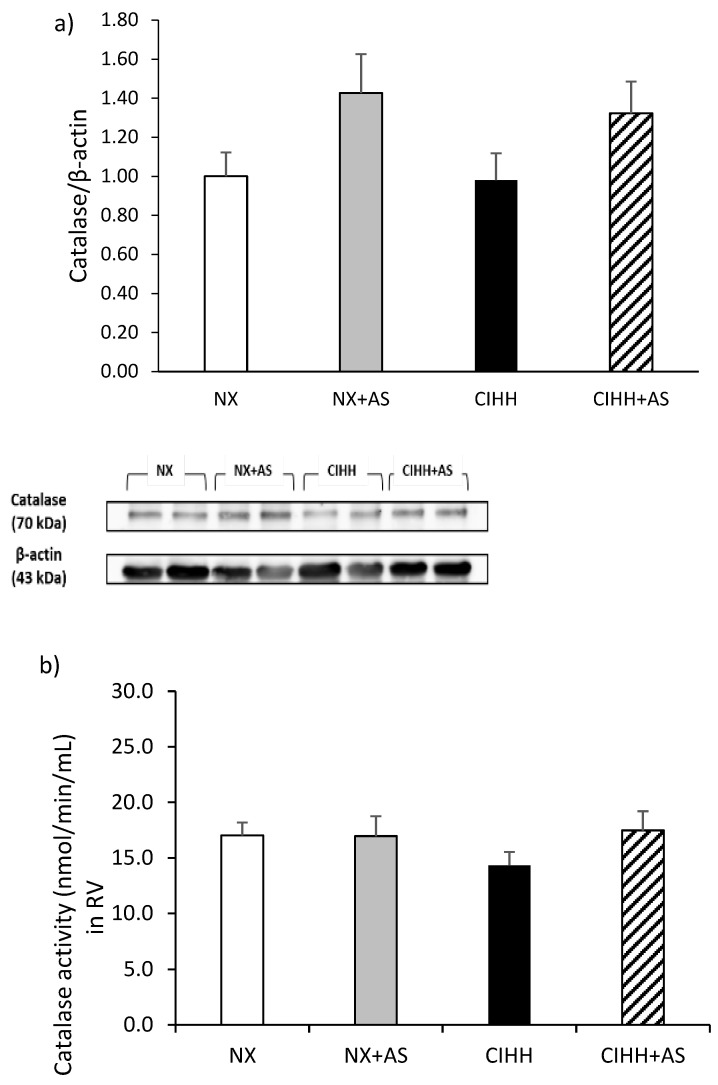

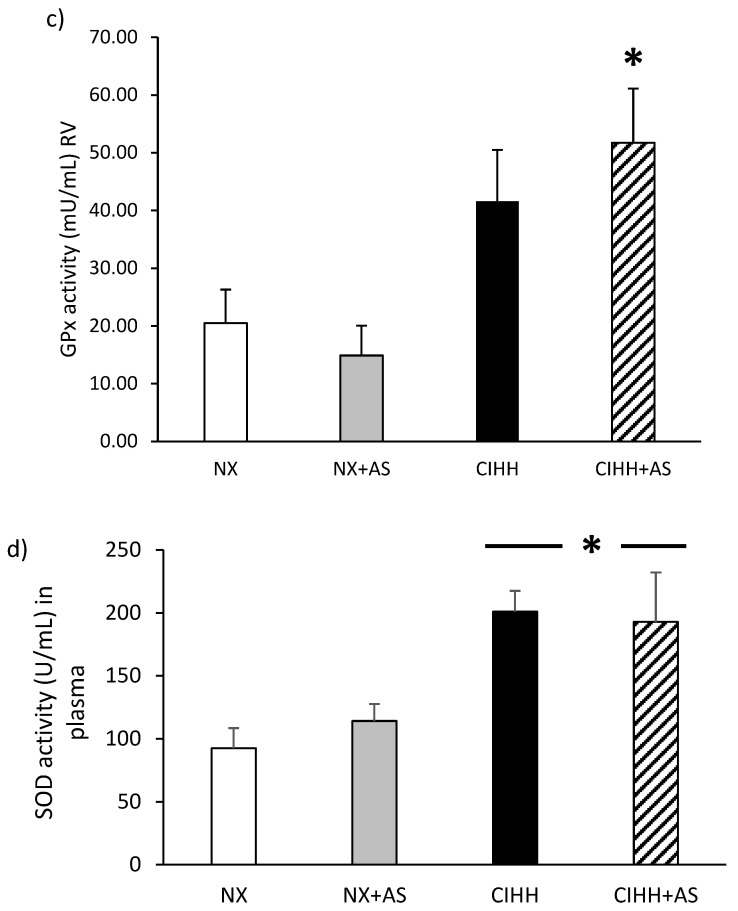
(**a**) Expression of catalase (CAT) in the right ventricle (RV); (**b**) CAT activity in the RV tissue; (**c**) glutathione peroxidase (GPx) activity in the RV; (**d**) superoxide dismutase (SOD) activity in the plasma of rats. The normobaric normoxia (NX), normobaric normoxia plus astaxanthin administration (NX + AS), chronic intermittent hypobaric hypoxia (CIHH), and chronic intermittent hypobaric hypoxia plus astaxanthin administration (CIHH + AS) groups were compared. The values are the means (x^−^) ± standard errors of the means (SEMs). * *p* < 0.05 hypobaric hypoxia groups vs. normoxia groups.

CAT protein levels and activity in the RV did not significantly differ between the groups (*p* = *NS*; Figure 5a,b). On the other hand, the activity of GPx in the RV was significantly increased only in the CIHH + AS group (*p* < 0.05; Figure 5c). In addition, SOD activity in the plasma increased in both hypoxic groups (*p* < 0.05; Figure 5d).

### 3.6. Inflammation

**Figure 6:**
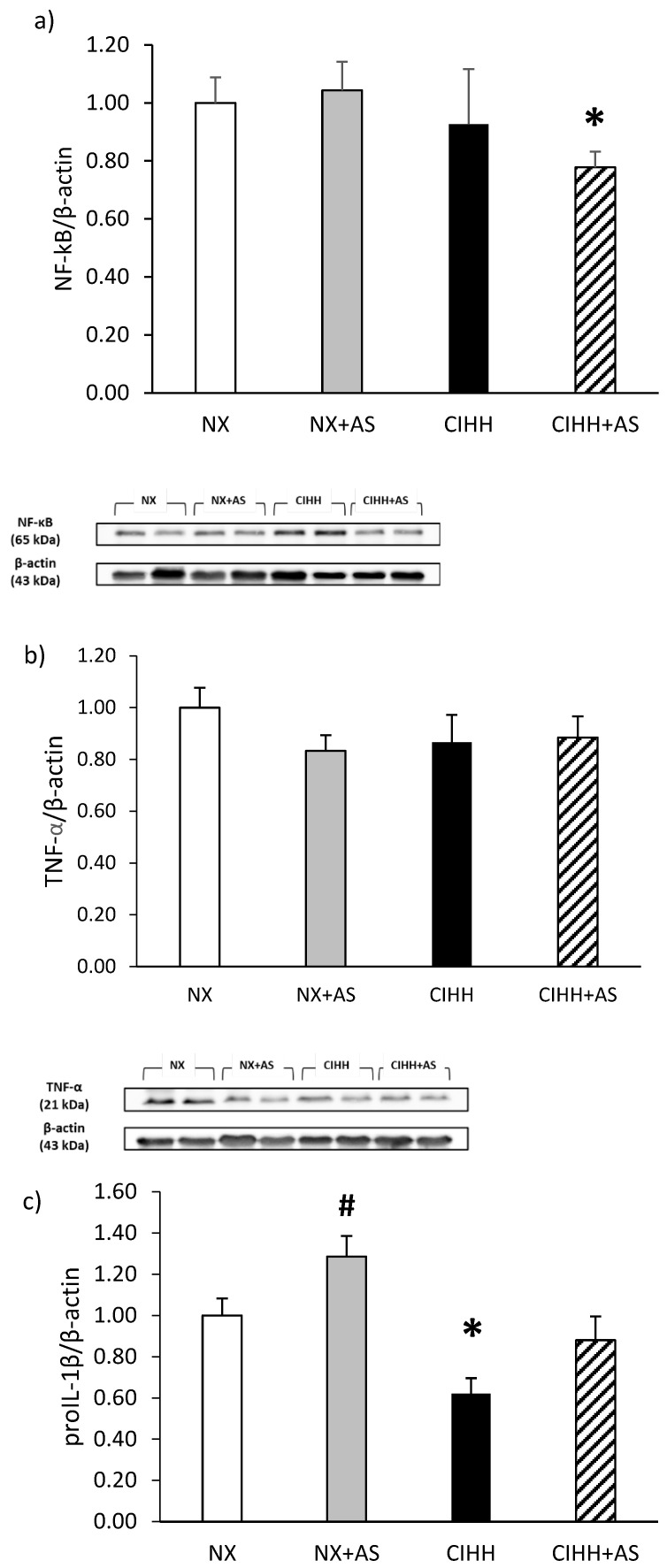

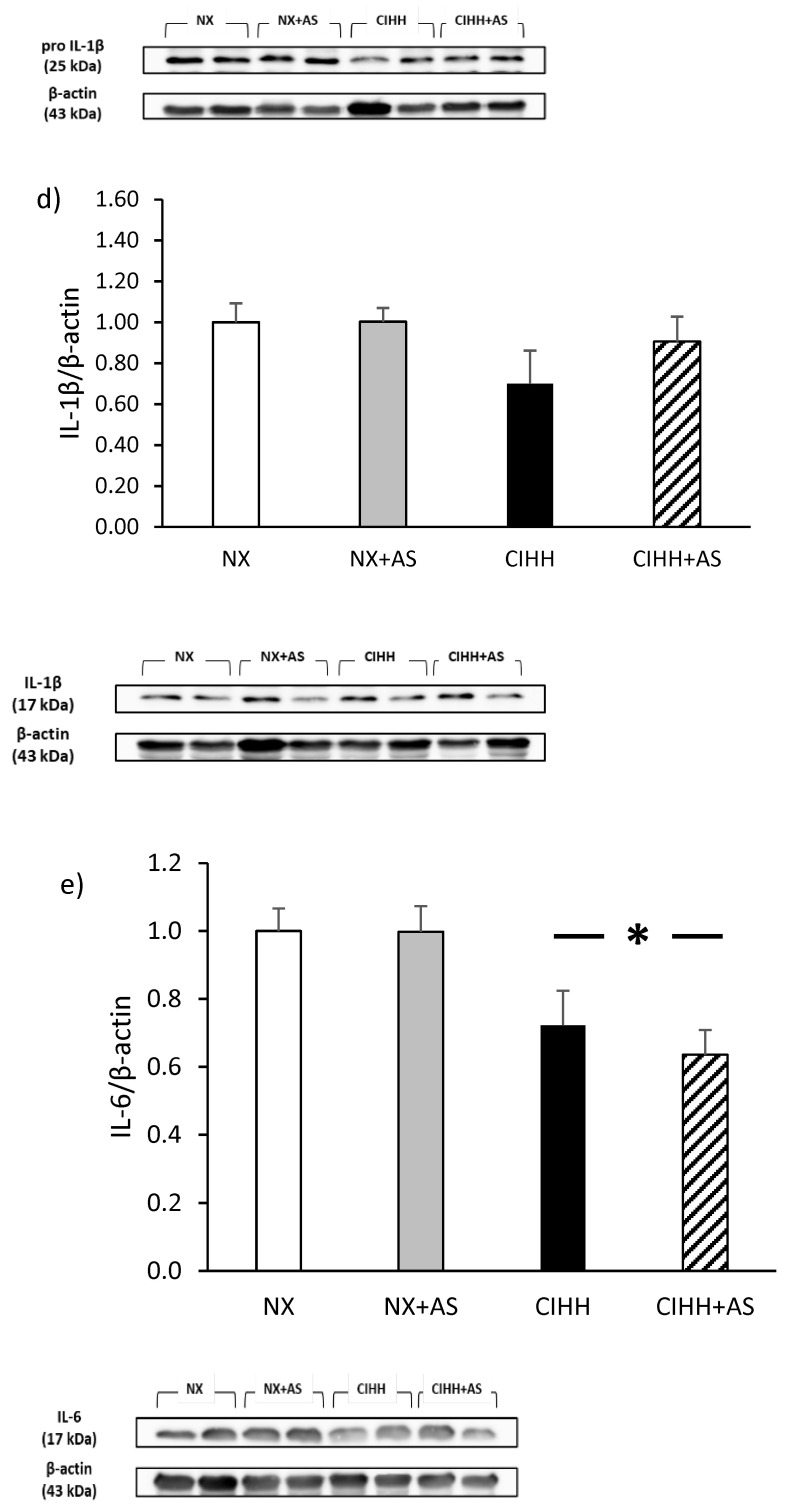
Expression of (**a**) nuclear factor kappa B (NF-kB); (**b**) tumor necrosis factor (TNF-α); (**c**) pro-interleukin-1b (pro-IL-1b); (**d**) interleukin-1β (IL)-1β, and (**e**) interleukin (IL)-6 in the right ventricle (RV) of rats in the normobaric normoxia (NX), normobaric normoxia plus astaxanthin administration (NX + AS), chronic intermittent hypobaric hypoxia (CIHH), and chronic intermittent hypobaric hypoxia plus astaxanthin administration (CIHH + AS) groups. The values are the means (x^−^) ± standard errors of the means (SEMs). * *p* < 0.05, hypobaric hypoxia group vs. NX group; # *p* < 0.05 NX-AS vs. NX group.

The protein expression of NF-kB in RV showed a decreased level in the astaxanthin groups compared to the control group (*p* < 0,05; Figure 6a); in the case of TNF-α expression in RV, tissues did not differ among any of the study groups (*p* = *NS*; Figure 6b). However, pro-interleukin-1b (pro-IL-1β) expression decreased significantly in the CIHH group, with astaxanthin administration returning it to the levels in the normoxic control group (Figure 6c), however, the IL-1β expression did not show differences in any group of study (*p* = *NS*; Figure 6d). Surprisingly, IL-6 levels were lower in both hypoxic groups than in the normoxic control group (*p* < 0,05; Figure 6d).

## 4. Discussion

In this study, we aimed to identify a new approach for treating right ventricular hypertrophy induced by CIHH. The results of this study show that exposure to CIHH induces RVH and increases lipid peroxidation and Nox2 expression, in addition to increasing SOD and GPx antioxidant activity. Moreover, the administration of astaxanthin returns oxidative stress markers (Nox2 expression and MDA levels) to the values observed under normoxic control conditions and ameliorates RVH, highlighting the increase in the antioxidant activity of GPx under exposure to CIHH + AS.

This research revealed a significant decrease in BW on day 29; such a decrease in BW under chronic and intermittent hypobaric hypoxia has been well described [29,34]. Weight loss could be attributed to a reduction in appetite, since studies have shown that high-altitude exposure increases the level of leptin; moreover, the duration of hypoxia could also determine the anorexigenic effect of the condition [35]. Furthermore, high-altitude exposure contributes to gastrointestinal malabsorption and an increase in the basal metabolic rate [36]. We also observed a gain in body weight in rats exposed to CIHH on day 24; however, this was followed by a decrease. These effects could be explained by the acclimatization period to hypoxia. After that, the pathologies associated with high altitude emerged, resulting in further weight loss in the rat groups, a phenomenon that has been discussed in previous studies, where mortality of this kind of exposure reached 40%, which was considered as a mortality predictor for this type of hypoxia [29].

Nevertheless, the rats treated with astaxanthin during exposure to CIHH presented with less severe BW loss than those subjected to hypoxia but not treated with astaxanthin. Thus, we suggest that the natural antioxidant astaxanthin can diminish the severity of AMS symptoms (i.e., nausea, vomiting, headache, and fatigue), as has been shown in previous studies involving the administration of vitamins such as L-ascorbic acid, tocopherol acid, and alpha-lipoic acid [37]. In other words, in this study, the rats might have experienced less severe AMS symptoms and thus consumed more food due to the antioxidant properties of astaxanthin. In addition, studies have shown a reduction in nutrient levels due to diminished intestinal absorption associated with oxidative stress [38], which could be alleviated by astaxanthin.

The first finding was an increase in the Hct% and the incidence of RVH in the CIHH group, consistent with previous reports [34,39,40], highlighting that an increase in the Hct% can result in viscosity that can contribute to the development of cardiac hypertrophy [41]. Notably, both BW and Hct% have been used as mortality predictors in studies using similar animal models and intermittent hypobaric hypoxia conditions [29].

Studies of cardiac tissue have shown that oxidative stress and inflammation occur simultaneously and are responsible for the activation of molecular pathways in cardiac hypertrophy and failure [42]. Importantly, previous studies have demonstrated that the RV is more susceptible to oxidative stress than the left ventricle is [43,44]; moreover, previous studies have reported an increase in oxidative stress [15] and inflammation [14] in RVH and right heart failure induced by hypobaric hypoxia. In this study, we confirmed an increase in Nox2 expression and MDA levels in the RV of rats exposed to CIHH, where the administration of astaxanthin decreased lipid peroxidation (MDA levels) and Nox2 expression, highlighting that these molecules play important roles in the development of oxidative stress, which will be discussed in the following paragraph. These antioxidant effects are supported by a recent study showing that the administration of astaxanthin to cultured cardiac cells decreases the hypertrophic effects of angiotensin II by decreasing the expression of GATA binding protein 4 (GATA4), brain natriuretic peptide (BNP), and atrial natriuretic peptide (ANP) [45], among which GATA4 can be activated by ROS [46].

Nox2 is an important source of ROS and contributor to oxidative stress in cardiac tissue [43], and our previous study demonstrated overexpression of Nox2 and increased lipid peroxidation in RVH induced by CIHH [15]. In the present study, an increase in the levels of Nox2 and lipid peroxides was also associated with oxidative stress; however, the administration of astaxanthin decreased the expression of Nox2 and lipid peroxidation and ameliorated RVH. This suggests that CIHH-induced RVH is associated more strongly with oxidative stress than with inflammation since an increase in the levels of inflammatory molecules was not observed under CIHH.

Under hypobaric hypoxia, the activity of GPx in the RV showed a non-significant increase; however, upon the administration of astaxanthin, the activation of this antioxidant enzyme was significantly potentiated, which could be related to the decrease in the levels of oxidative stress biomarkers in the RV. Studies have shown that GPx and SOD play a key role in cardiomyocytes and the intrinsic heart antioxidant system associated with cardiac hypertrophy and remodeling [16,47]. Additionally, a human study revealed increased GPx activity in the plasma under hypobaric hypoxia exposure compared with normobaric hypoxia exposure [13]; therefore, these results corroborate the important role of oxidative stress in the development of RVH induced by CIHH, which can be mitigated by the administration of the antioxidant astaxanthin.

With respect to inflammation, exposure to acute hypobaric hypoxia sensitizes monocytes and T cells, which release TNF-α, IL-1α, and IL-1β; however, in this study, CIHH did not alter the level of TNF-α [48]. Moreover, the results of this study revealed a decrease in pro-IL-1β levels in CIHH, whereas the administration of astaxanthin increased the level of this proinflammatory cytokine to control levels; therefore, based on recent studies that showed that astaxanthin administration inhibits the NLRP3 inflammasome and caspase-1 activity, while decreasing the activation and secretion of IL-1β in mice [49], therefore, a possible explanation of the present study could be that the astaxanthin treatment contribute to the pro-IL-1β accumulation, preventing the cleavage to the active form of this cytokine.

Surprisingly, the level of IL-6 was decreased in the RV of rats exposed to CIHH, which is inconsistent with several studies showing an increase in the level of this cytokine in the RV under hypobaric conditions [50]. However, these results could be due to the intermittent nature of the exposure conditions used in this study since a recent study by Shati and colleagues [51] revealed rapid restoration of the level of this cytokine (3 h) in the hearts of rats after exposure to hypobaric hypoxia (5000 m). On the other hand, a study involving an experimental animal model of hypoxia [52] revealed increased expression followed by decreased expression of IL-6 under hyperoxic conditions, demonstrating an association between the IL-6 level and the frequency of intermittent hypoxia, which is congruent with NF-kB expression in this study.

Importantly, previous studies have shown that IL-6 plays a role in the acute phase of hypoxia [53], in which IL-6 is associated with the effects of hypoxia on erythropoietin (EPO) stimulation but not inflammatory activity [54]; this could also explain the results related to IL-6 levels in the RV of rats subjected to CIHH, on which the administration of astaxanthin had no significant effect. Additionally, a recent study revealed that intermittent hypoxia exerts an anti-inflammatory effect by increasing the levels of ROS produced by Nox2, which activates transcription factors that activate antioxidant and anti-inflammatory signaling pathways [50]; this mechanism is relevant because Nox2 expression was increased in our studies, however more studies are needed to clarify this statement.

Although in this study we did not analyze the mechanism by which astaxanthin exerts its protective effects, other authors have investigated the pathways that may be involved, for example, various studies highlight astaxanthin as a powerful antioxidant that can activate the sirtuin pathway, particularly SIRT1. This pathway regulates key cellular processes, such as enhancing mitochondrial function, reducing ROS, and modulating genes associated with longevity and cellular stress. This mechanism indicates a potential pathway through which astaxanthin mediates its beneficial effects [55]. Additionally, an interesting study demonstrated that astaxanthin administration improved cardiac function and attenuated fibrosis, which was linked to increased expression and activity of SIRT1 [56]. This finding is supported by studies showing that the astaxanthin administration elevated levels of antioxidants (glutathione, catalase, and manganese superoxide dismutase) and reduced levels of ROS, TNF-α, and IL-6, mediated through SIRT1 and the Keap1-Nrf2 signaling pathway in cardiac tissue [57,58]. It is also important to note that astaxanthin can exert a protective effect in myocardial cells by decreasing HIF-1α stabilization via miR-1338 [23]. Therefore, the protective mechanisms of astaxanthin in the heart may involve the Nrf2 and SIRT1 pathways.

## 5. Conclusions

This study revealed a notable increase in the level of the oxidative stress biomarker MDA, increased expression of Nox2, a source of ROS, a decrease in pro-IL-1β levels, and the presence of right ventricular hypertrophy in rats exposed to chronic intermittent hypobaric hypoxia (CIHH). However, the administration of astaxanthin notably alleviated RVH, decreased oxidative stress markers (Nox2 expression and the MDA concentration), increased the activity of GPx, and increased the expression of pro-IL-1β in the RV of rats subjected to CIHH. Therefore, astaxanthin could be an important cardiac protector under hypoxic conditions. This study opens a new avenue for understanding the mechanisms of hypobaric hypoxia and the antioxidant properties of astaxanthin as a new nutraceutical treatment.

## Data Availability

Data contained within the article.
